# Oral Transmucosal or Intramuscular Administration of Dexmedetomidine–Methadone Combination in Dogs: Sedative and Physiological Effects

**DOI:** 10.3390/ani10112057

**Published:** 2020-11-06

**Authors:** Daniela Gioeni, Federica Alessandra Brioschi, Federica Di Cesare, Vanessa Rabbogliatti, Martina Amari, Sergio Zanzani, Petra Cagnardi, Giuliano Ravasio

**Affiliations:** 1 Department of Veterinary Medicine, Università degli Studi di Milano, 20122 Milan, Italy; daniela.gioeni@unimi.it (D.G.); federica.brioschi@unimi.it (F.A.B.); sergio.zanzani@unimi.it (S.Z.); petra.cagnardi@unimi.it (P.C.); giuliano.ravasio@unimi.it (G.R.); 2 Department of Health, Animal Science and Food Safety, Università degli Studi di Milano, 20122 Milan, Italy; 3 Department of Veterinary Medicine, Centro Clinico Veterinario e Zootecnico Sperimentale, Università degli Studi di Milano, 20122 Milan, Italy; vanessa.rabbogliatti@unimi.it (V.R.); martina.amari@hotmail.it (M.A.)

**Keywords:** anesthesia, dexmedetomidine, dog, intramuscular, methadone, oral transmucosal, sedation

## Abstract

**Simple Summary:**

During the last decade, new alternative non-invasive administration routes for drug delivery have gained interest in veterinary medicine. The administration of drugs via the oral transmucosal route is non-invasive, painless, easy to perform, and generally well tolerated. Furthermore, it avoids gastric acid degradation typical of oral administration. All these characteristics contribute to make this administration route very attractive, especially for veterinary patients who are difficult to inject, fearful, or anxious. In contrast, intramuscular injection is associated with pain and requires more invasive restraint, potentially incrementing patients’ discomfort. The aim of this study was to assess and compare the sedative and clinical effects of a dexmedetomidine–methadone combination following either oral transmucosal and intramuscular administration in healthy dogs and to record any possible adverse effects following each administration route. The present study suggests that oral transmucosal administration of dexmedetomidine and methadone combination provided a satisfactory level of sedation, allowing safe handling of the patients with less pronounced cardiorespiratory effects. Indeed, thanks to the lesser impact on the cardiac function, it could be considered as a useful option for those patients difficult to restrain in which cardiovascular stability should be preserved.

**Abstract:**

The aim of this study was to compare the sedative and physiological effects following either oral transmucosal (OTM) or intramuscular administration of dexmedetomidine–methadone combination in healthy dogs. Thirty dogs were randomly assigned to receive a dexmedetomidine–methadone combination either by the OTM (*n* = 15) or intramuscular (*n* = 15) route. Sedation was scored 10, 20, and 30 min after drugs administration. Heart rate (HR), non-invasive blood pressure (NIBP), respiratory rate (f_R_), and body rectal temperature were recorded before drugs administration and then every 10 min for 30 min. Propofol dose required for orotracheal intubation was recorded. Sedation scores increased over time within both groups with higher values in intramuscular group (*p* < 0.05). Within each group, HR decreased significantly compared with baseline *(p* < 0.001) and was significantly lower in intramuscular group compared with the OTM group (*p* < 0.001). In both groups, NIBP increased significantly compared with baseline (*p* < 0.05). In the intramuscular group, f_R_ was lower compared with the OTM group at all the observational time points (*p* < 0.001). Propofol dose was lower in the intramuscular group (*p* < 0.05). Compared to intramuscular dexmedetomidine–methadone, OTM combination produced lower but effective sedation in healthy dogs.

## 1. Introduction

During the last decade, new alternative non-invasive administration routes for sedative and analgesic drugs have gained interest in veterinary medicine [1,2,3,4]. The drugs administration via the oral transmucosal (OTM) route allows rapid systemic absorption by the oral cavity membrane due to its rich blood supply [5]. Moreover, OTM drug delivery is reported to be non-invasive, painless, easy to perform, and generally well tolerated [6]. Furthermore, it avoids gastric acid degradation and first-pass liver metabolism of drugs, typical of oral administration [7]. All these characteristics contribute to make this administration route very attractive, especially for patients who are difficult to inject, fearful, or anxious [2,8,9,10].

Drug delivery by the OTM route has been widely studied in cats, both from a clinical and a pharmacokinetic point of view. Opioids, administered via the OTM route alone or combined with dexmedetomidine, represent the most studied molecules in this species [5,6,7,11,12,13]. In dogs, OTM administration of α_2_-adrenoreceptor agonists [2,14,15] and opioids [4,16,17] have been described. However, only one study reported the pharmacokinetic profile of these two drug classes administered in combination via the OTM route [18].

Dexmedetomidine, the most selective α_2_-adrenoreceptor agonist, is commonly used in small animal practice to provide sedation, analgesia, and muscle relaxation [19]. On the other hand, dexmedetomidine causes dose-related cardiovascular changes, such as a powerful but transitory peripheral vasoconstriction, bradycardia, and reduction in the cardiac output [19].

Methadone is a synthetic full μ-agonist opioid presenting analgesic potency and pharmacokinetic characteristics similar to those of morphine. Still, it also has antagonistic activity on *N*-methyl-d-aspartate (NMDA) receptors [20] and inhibitory effects on the reuptake of noradrenaline and serotonin [21]. Methadone administration has been reported to produce a potent analgesic effect in dogs, together with dose-dependent sedation and cardiorespiratory effects [22,23].

Alpha_2_-adrenoreceptor agonists and opioids co-administration potentiates their sedative and analgesic effect, allowing a dose reduction in each drug and therefore reducing the cardiovascular effects mainly induced by α_2_-adrenoreceptor agonists [24,25]. In fact, α_2_-agonist and opioids work in synergism acting on the same signal transduction system (G-protein activation), because they have a partially overlapping central receptor localization [19].

To the authors’ knowledge, no studies have compared the sedative and physiological effects of a dexmedetomidine–methadone combination administered by either OTM or intramuscular route, in healthy dogs. Thus, this study aimed to assess and compare the sedative effects of a dexmedetomidine–methadone combination following either OTM or intramuscular administration in dogs and to record any possible adverse effect following each administration route. The hypothesis of the study was that the administration of dexmedetomidine–methadone combination would produce a satisfactory level of sedation in both OTM and IM (intramuscular) group. It was also hypothesized that OTM administration was able to induce less pronounced cardiovascular effects.

## 2. Materials and Methods

The study protocol was approved by the Institutional Ethical Committee for Animal Care at the University of Milan (OPBA_19_2016). All dogs were enrolled after obtaining owners’ written consent.

### 2.1. Animals

Based on the results of previous reports [24], we estimated that a minimum of 13 dogs per group would be necessary to attain a 95% confidence level of achieving a clinically significant difference (at least 20%) in heart rate (HR) between groups with 80% power.

Thirty client-owned dogs, older than six months and younger than 96 months of age, of any breed, gender, and bodyweight, needing sedation before general anesthesia for elective surgeries or diagnostic procedures were enrolled in the study. The dogs included were considered healthy (American Society of Anaesthesiology class I) based on medical history, physical examination, complete blood count, serum biochemical analysis, and echocardiographic examination. Animals receiving any medication within 30 days prior to surgery or diagnostic procedure or with any oral cavity disease or alteration were excluded from the study.

### 2.2. Study Design

A prospective randomized blinded clinical trial was designed and then completed within a six-month period. Randomization was achieved via a computer-generated random sequence using a commercial software program (Microsoft Office Excel 2013; Microsoft Corp, Redmond, WA, USA). All dogs were fasted for 12 h, and water was withheld for two hours before the beginning of the study. A temperament evaluation was carried out in all dogs using a score ranging from 0 (very relaxed) to 4 (very excitable/nervous) [26]. The animals were acclimatized to the environment in a quiet room for approximately 20 min prior to administration of any treatment. Subsequently, dogs were randomly premedicated with a drug mixture containing 10 µg kg^−1^ of dexmedetomidine hydrochloride (Dexdomitor^®^ 0.5 mg mL^−1^, Vetoquinol, Bertinoro, Italy) and 0.4 mg kg^−1^ of methadone hydrochloride (Semfortan^®^ 10 mg mL^−1^, Dechra Veterinary Products, Torino, Italy) either via the OTM route (OTM group) or via the intramuscular route (IM group). Drugs were mixed in the same syringe just before administration. Oral transmucosal administration was performed by inserting the nozzle of a 2.5 mL syringe into the dogs’ buccal pouch, and the content was splashed into the cheek area. In the IM group, drugs were injected into the epaxial muscles after verifying no intravascular injection. In the OTM group, the total volume of drugs administered was recorded, and oral pH was measured before and 10 min after drugs administration.

The degree of sedation was assessed with a modified numeric rating scale adapted from Gurney et al., 2009 [27], ranging from 0 (lack of sedation) to 13 (maximum sedation), as showed in Table 1.

Sedation score was recorded at 10 (T10), 20 (T20), and 30 (T30) minutes after drugs administration. The following variables were monitored during the observational period (30 min): HR obtained from a continuous lead II electrocardiography recording, systolic, mean, and diastolic non-invasive arterial blood pressures (SAP, MAP; DAP), using a high definition oscillometric device (Memo Diagnostic HDO Pro, S+B MedVet GmbH, Babenhausen, Germany), respiratory rate (f_R_) obtained by observation of thoracic excursions, and body rectal temperature (BT) recorded with a digital thermometer. For non-invasive blood pressure (NIBP) monitoring, the cuff was positioned around the antebrachium, proximal to the carpus. The cuff selected was provided with the device according to the manufacturer’s instructions. During measurements, five consecutive readings were obtained. The highest and lowest values were excluded, and the mean value was used for analysis [28]. Physiological parameters (HR, SAP, MAP, DAP, f_R_, BT) were recorded before (baseline, T0) and at 10 (T10), 20 (T20), and 30 (T30) minutes after drugs administration. At T30, a peripheric venous catheter (20 gauge; Delta ven 1, Delta Med, Viadana, Italy) was aseptically placed into the right cephalic vein, and the patients’ resistance to venous catheterization was scored using a scale ranging from 0 (strong resistance) to 3 (no resistance) [29]. Then, general anesthesia was induced in all dogs by titration of intravenous propofol (Proposure, 10 mg mL^−1^, Merial Italia S.p.A., Milan, Italy) to achieve orotracheal intubation. The dose of propofol required for intubation was recorded. During the observational period, the appearance of any adverse event such as severe bradycardia, arrhythmias, excitement, ptyalism, or vomiting was recorded. In the case of severe bradycardia or bradyarrhythmia (HR less than 34 bpm or third-degree atrioventricular block), atipamezole (50 µg kg^−1^) was administered intravenously and dogs were excluded from further assessments. Data recorded after induction of general anesthesia were not considered in the present study.

All the sedation score assessments and the physiological variables measurements were performed by an experienced anesthetist unaware of the drugs delivery route. The evaluation of the oral pH was performed by the same anesthetist who administered the drugs through a litmus pH paper strip (range from 7 to 14), by inserting the strip into the dogs’ buccal pouch.

### 2.3. Statistical Analysis

Statistical analysis was performed using the dedicated software IBM SPSS Statistics 20.0 (SPSS Inc., Chicago, IL, USA). Differences with *p* < 0.05 were considered significant.

Normality of data distribution was assessed by a Shapiro–Wilk test at the α = 0.05 level. Descriptive statistics were reported as mean and standard deviation or median and range for continuous and categorical variables, respectively.

Variables normally distributed were compared between treatment groups by using Student’s t-test (temperament and venous catheterization) or with a two-way repeated-measures ANOVA (HR SAP, MAP, DAP, f_R_, and BT) followed by the Bonferroni post hoc test and compared within groups with an adjusted multiple comparison test.

A generalized linear mixed model (GLMM) including the fixed effects of administration route, time since administration, administration route × time since administration was also applied to the sedation score. The structures for GLMM were chosen based on the best model fit and smallest value of corrected Akaike Information Criterion.

Finally, a univariate analysis was performed on the dose of propofol required to achieve orotracheal intubation to assess the influence of the administration route. Further, all variables were entered in a multivariate model, developed by backward elimination until all remaining variables were significant.

## 3. Results

All dogs enrolled (*n* = 30) successfully completed the study. Fifteen dogs were randomly assigned to the OTM group and 15 dogs to the IM group. No statistical differences (*p* > 0.05) between the OTM and IM groups were observed for age (45 ± 29 months and 46 ± 29 months, respectively) and weight (26 ± 6 kg and 27 ± 4 kg, respectively). Mixed breed was equally overexpressed (eight dogs in each group), and also, sex distribution was comparable between groups (seven males and eight females in the OTM group; eight males and seven females in the IM group). No statistical difference (*p* > 0.05) was observed between groups for the temperament score (OTM median 2, range 1–3 and IM median 2, range 1–4). The volume of drugs mixture administered in the OTM group was 1.6 ± 0.4 mL. Within the OTM group, there was no significant difference in oral pH, measured before and at 10 min after drugs administration (9.0 ± 0.5 and 8.9 ± 0.4, respectively).

The median sedation scores are presented in Table 2. There was a significant effect of the administration route (*p* < 0.001), time since administration (*p* < 0.001), and administration route × time since administration (*p* < 0.001) on sedation score.

**Figure 1:**
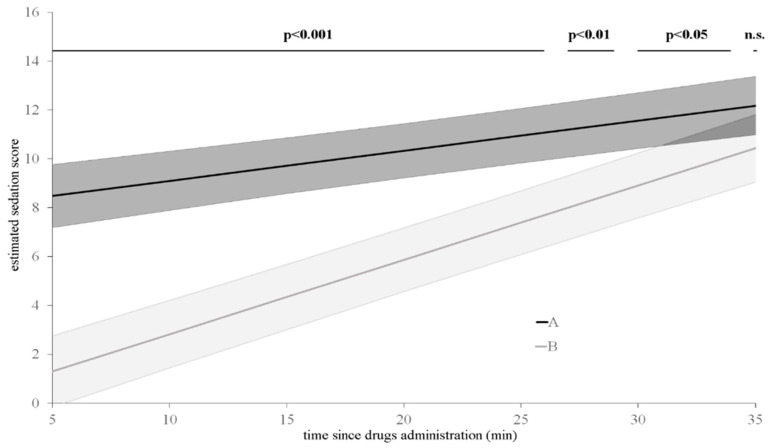
Sedation scores estimated by the model in oral transmucosal (grey line) and intramuscular (black line) treated dogs. Black bars: significance of pairwise comparisons between oral transmucosal and intramuscular administration; n.s.: not significant.

Furthermore, the model confirmed that, compared with the OTM group, the sedation score in the IM group increased earlier and presented higher values throughout the evaluation period despite the level of significance decreasing over time (*p* < 0.001 until T26; *p* < 0.01 between T26 and T28; *p* < 0.05 between T28 and T34). Finally, as resulted from the predictive simulation test extrapolated by the GLMM, the difference in sedation score between groups disappeared 35 min after drugs administration (*p* > 0.05) (Figure 1).

The mean values of all the physiological variables are also presented in Table 2. There were no statistical differences between the OTM and IM groups in baseline values for any of the physiological variables evaluated. Within each treatment group, HR was lower at all time points (T10, T20, and T30) compared with T0 *(p* < 0.001). Moreover, within both groups, there was a significant decrease in HR over time between T10 and T20 and T10 and T30 (*p* < 0.001), with a further difference within the OTM group between T20 vs. T30 (*p* < 0.01). Between groups, HR was significantly lower in the IM group compared with the OTM group at all the observational time points (*p* < 0.001).

In the OTM group, SAP and MAP were higher at T30 compared with T0 (*p* < 0.05) and T10 *(p* < 0.05), while DAP presented higher values only at T30 compared with T0 (*p* < 0.05). Differently, in the IM group, SAP and MAP were higher at T10 and T20 compared with T0 (*p* < 0.05) and T30 (*p* < 0.01), while DAP was higher at T10 and T20 compared with T30 (*p* < 0.01). Between groups, SAP, MAP, and DAP were higher at T10 and T20 in the IM group compared with the OTM group (*p* < 0.05).

Within each treatment group, f_R_ was lower at all the observational time points (T10, T20, and T30) compared with baseline (OTM group: T0 vs. T10 *p* < 0.05, T0 vs. T20, and T0 vs. T30 *p* < 0.01; IM group: T0 vs. T10, T0 vs. T20, and T0 vs. T30 *p* < 0.001). Within the OTM group, there was a decrease in f_R_ over time (T10 vs. T20 *p* < 0.05, T10 vs. T30 *p* < 0.01 and T20 vs. T30 *p* < 0.05). Between groups, f_R_ was lower in the IM group compared with the OTM group at all the observational time points (*p* < 0.001).

Regarding BT, although there was a slight decrease in body temperature over time, this was not significant within each group and between groups.

For venous catheterization, no difference was detected between groups (*p* > 0.05). The score of 3 (no resistance) was observed in 100% and 87% of dogs in the IM and OTM groups, respectively. The remaining 13% of dogs in the OTM group obtained a score of 2 (moderate resistance).

There was a significant effect of the administration route on the dose of propofol required to obtain orotracheal intubation that was significantly lower in the IM group (1.6 ± 0.8 mg kg^−1^) compared with the OTM group (2.9 ± 0.8 mg kg^−1^) (*p* < 0.05).

Regarding the possible adverse effects related to the different routes of administration, four out of 15 dogs receiving intramuscular treatment exhibited second-degree atrioventricular block throughout the observational period but did not require any treatment. In the OTM group, ptyalism was recorded in eight out of 15 dogs (53%) and emesis in two out of 15 dogs (13%).

## 4. Discussion

Nowadays, due to the growing interest in minimizing patient discomfort as much as possible in a clinical setting, unconventional systemic routes of drugs administration, such as OTM or intranasal, represent interesting alternatives to the intramuscular or intravenous drug delivery in dogs [1,14,30]. Intranasal and OTM administrations are painless, easy to perform with minimal patient restraint, and generally well tolerated, even in aggressive animals [2]. In contrast, intramuscular injection is associated with pain [31] and requires more invasive restraint, potentially incrementing patients’ discomfort [8]. Furthermore, OTM administration promotes rapid systemic drugs absorption and avoids hepatic first-pass metabolism and gastrointestinal degradation [14].

To the authors’ knowledge, no study has investigated the clinical profile of dexmedetomidine–methadone combination through the OTM route.

The simultaneous administration of 10 µg kg^−1^ of dexmedetomidine and 0.4 mg kg^−1^ of methadone via the OTM route induced satisfactory level of sedation, allowing safe patient management and easy placement of venous catheter. Although, the time needed to obtain an appropriate level of sedation was longer and scores achieved were lower compared with the intramuscular route. The onset of sedation was detected between 15 and 20 min with the highest score recorded at T30 in the OTM group, while dogs treated with the intramuscular dexmedetomidine–methadone combination showed high sedation scores within 10 min, with only a mild increase between T10 and T30. Dent and colleagues (2019) [14] and Cohen and Bennet (2015) [2] described similar findings in dogs following OTM administration of dexmedetomidine alone. Both studies reported the onset of sedation between 15 and 30 min after OTM administration, even in aggressive dogs [2]. In the present study, a mild/deep sedation was achieved with a lower dose of dexmedetomidine compared with the studies mentioned above, probably due to the co-administration with methadone. This finding is supported by the literature, in which the synergistic effect between α_2_-adrenoreceptor agonists and opioids is reported [25].

Oral transmucosal absorption of drugs is dependent on a number of predictable and unpredictable factors. Predictable factors include the molecular size and chemical behaviour of the drugs, partition coefficient of the drugs, inherent properties of the oral mucosa, and drugs concentration [14]. Dexmedetomidine and methadone are relatively small lipophilic drugs, with a pKa of 7.1 and 8.9, respectively, and as weak base drugs, they are preferentially unionized at increasing pH, which enhances their ability to cross the oral mucosa and facilitate absorption [14]. In the present study, the oral pH alkalinity (8.8 ± 0.4) could have favored the presence of a unionized form, increasing the drugs absorption [4,11]. The relationship between the pH and pKa of the drug combination and the pH of the oral mucosa was indirectly investigated by measuring the pH of the oral cavity of dogs before and after the administration of the drugs. Regardless of the pH of the drug combination and the pKa of dexmedetomidine and methadone, the dogs’ pH of the oral cavity was not significantly different before and after OTM administration. This fact seems to further support the idea that the pH of the drug combination is unlikely to have played an important role in influencing the bioavailability of OTM administration. The pH of dexmedetomidine and methadone (alone and in combination) was not measured as both drugs used in this study are injectable formulations registered for intravenous and intramuscular use, which means that both active ingredients are dissolved in a buffer solution. Consequently, the combination of the two drugs could not have presented a substantially different pH than dexmedetomidine and methadone alone. Similarly, the pKa of the drugs would not change within the buffer solution, continuing to favor the unionized (and more easily absorbed) form of the two active ingredients. These considerations support the idea that the pH of the drug combination is unlikely to have played an important role in influencing the bioavailability of OTM administration.

However, OTM absorption is also influenced by different unpredictable effects that can explain the high level of variability between subjects. Ptyalism, observed in 8/15 dogs in the OTM group and the partial loss (outside the mouth) or the swallowing of drugs during administration are associated with decreased systemic drugs bioavailability, as previously described in dogs [14,18] and in cats [10]. Moreover, the administered volume, strictly related to drugs formulation and concentration, plays an important role concerning the potential drug loss. In fact, smaller volumes have less chance of inducing swallowing and/or loss of drugs outside the mouth resulting clinically more effective [9,10]. In the present study, it was decided to simultaneously administer the injectable formulation of both dexmedetomidine and methadone, and this resulted in a relatively high total mixture volume administered (1.6 ± 0.4 mL). Particularly, methadone injectable formulation presented a lower concentration compared with other drugs, such as dexmedetomidine, consequently increasing the mixture volume. Finally, another factor that may have compromised the systemic absorption of dexmedetomidine–methadone combination is the peripheral vasoconstriction produced by dexmedetomidine itself, which is normally induced by the interaction of the drug with the α-2B receptors of the precapillary sphincters of the peripheral vascular beds, which leads to a reduction in peripheral drug absorption [5,18]. These considerations, together with the other mentioned factors, could explain the higher variability between subjects in sedation score recorded in the OTM group.

A decrease in HR and an increase in NIBP variables over time by either OTM or intramuscular route compared with baseline values was observed. Dexmedetomidine-induced bradycardia is commonly reported in dogs after intravenous [32], intramuscular [33], and OTM [14] administration. Dexmedetomidine effects on the cardiovascular system are mainly due to its α_2A-B_-adrenoceptors agonism, which produces an increase in systemic vascular resistance and a centrally mediated bradycardia [34]. The administration of α_2_-agonists induces an initial, transient hypertensive phase accompanied by bradycardia that, in the present study, could be appreciated especially in the IM group. In fact, in this group, the decrease in HR and the increase in blood pressure appeared earlier and were more pronounced compared with the OTM group. Conversely, in the OTM group, HR decreased gradually and remained generally higher than in the IM group. Blood pressure in the OTM group became significantly higher compared with T0 only 30 min after drugs administration. As previously reported in different species, physiological effects obtained after OTM administration seem to be related to the drugs absorption that, for this route, is described to be slower and delayed compared with other routes, such as the intramuscular [5,18]. Furthermore, the cardiovascular changes associated with dexmedetomidine administration seem to be dose-related [19], therefore the simultaneous administration of methadone used in this study allowed a lower dexmedetomidine dose than reported in literature [2,14]. This dexmedetomidine dose reduction may have contributed to a mild and gradual onset of the effects on HR and blood pressure obtained in the OTM group. Respiratory rate decreased in both groups compared with T0. Dexmedetomidine and methadone, administered both via OTM and intramuscular routes, may have influenced f_R_. Data obtained in the present study support the findings reported in the literature that there is a reduction in f_R_ following the simultaneous intramuscular administration of dexmedetomidine and methadone. In fact, the combination of these two drugs may enhance the total respiratory depressant effect of each single drug [22]. Furthermore, in the IM group, f_R_ dropped to lower values faster and remained lower than in the OTM group for the entire observational period. This result needs to be interpreted in relationship with sedation scores. In general, dogs premedicated by intramuscular injection were more sedated compared with dogs receiving OTM administration. Dogs presenting a lighter level of sedation are probably more prone to be influenced by external stimulation that, especially in clinical setting, could cause an increase in f_R_ [35]. Conversely, in the OTM group, dogs showed a slower and more gradual decrease in f_R_. This finding could be explained considering the pharmacokinetic profile of the OTM route that reports a slower absorption and a lower bioavailability compared with the intravenous and intramuscular route [14,18].

Moreover, premedication with dexmedetomidine and methadone is reported to decrease propofol requirement for anesthesia induction compared with un-premedicated dogs [36] or to dogs premedicated with dexmedetomidine alone [37]. In the present study, dogs premedicated intramuscularly required a significant lower propofol dose (1.6 ± 0.8 mg kg^−1^) compared with the OTM group (2.9 ± 0.7 mg kg^−1^), and this could be explained considering the higher sedation score (in average) obtained in the IM group. Nevertheless, in the present study, OTM premedication with dexmedetomidine and methadone allowed orotracheal intubation with less propofol compared with results obtained by Davis and colleagues (2017) [36] in un-premedicated dogs.

The present investigation has some limitations correlated to the clinical nature of the study. First, the observational period was limited to 30 min, then general anesthesia was induced, and no further measurements were carried out. Nevertheless, according to the literature, it is possible that, also in this study, the peak of sedation in the OTM group appeared later than 30 min; Dent and colleagues (2019) [14] reported the highest level of sedation 38 min after dexmedetomidine OTM administration. Furthermore, although estimated by the model used in the present study, OTM sedation scores comparable to the IM group were expected 35 min after drugs administration. Nevertheless, the level of sedation obtained at T30 was considered in all dogs clinically satisfactory, allowing easy and safe patient handling and simple venous catheter placement. Moreover, the perioperative antinociceptive effect (especially for surgical patients) was not evaluated, nor was the recovery quality. Actually, the evaluation of the antinociceptive property after dexmedetomidine and methadone simultaneous administration via the OTM route in dogs could be an interesting aspect. In fact, in human medicine, this type of drug delivery is reported to provide a more effective and longer analgesia after surgery, compared with the intramuscular route [38].

## 5. Conclusions

In conclusion, in healthy dogs, the simultaneous administration of dexmedetomidine and methadone was effective to provide sedation after both OTM and intramuscular administration. Despite the fact that the level of sedation observed in the OTM group was slightly lower compared to the IM group, it was sufficient in allowing for safe patient handling and easy placement of a venous catheter. Furthermore, the OTM route appeared easy to perform and was well tolerated by the patients and characterized by less pronounced cardiorespiratory effects.

Indeed, thanks to the smoother impact on the cardiac function, oral transmucosal administration of dexmedetomidine and methadone combination could be considered as a useful option for those patients difficult to restrain in which the cardiovascular system is compromised.

## Figures and Tables

**Table 1:** Sedation modified numeric rating scale (adapted from Gurney et al., 2009 [27]).

Criteria	Description	Score
Spontaneous posture	Standing	0
	Sternally recumbent	1
	Laterally recumbent	2
Eye position	Forward (normal position)	0
	Rotated ventrally	2
Response to sound (handclap)	Body movement	0
	Head movement	1
	Ear twitch	2
	No reaction	3
Resistance to lateral recumbency	Full (stands)	0
	Moderate restraint required	1
	Mild restraint required	2
	No resistance	3
Overall appearance	No sedation apparent	0
	Mild sedation	1
	Moderate sedation	2
	Well sedated	3
Total possible sedation score		13

**Table 2:** Sedation scores and physiological variables over time in dogs administered dexmedetomidine (10 μg kg^−1^) and methadone (0.4 mg kg^−1^) via oral transmucosal administration (OTM group) or intramuscularly (IM group). Sedation scores are presented as median and interquartile range (IQR). Physiological variables are presented as a mean ± standard deviation. Main differences are indicated in the table. HR: heart rate; SAP: systolic arterial blood pressure; MAP: mean arterial blood pressure; DAP: diastolic arterial blood pressure; f_R:_ respiratory rate; BT: body rectal temperature.

Variable	Group	Time Points			
		T0	T10	T20	T30
*Sedation*	OTM	-	1 (4) ^†^	5 (5) ^†^	9 (5) ^†^
	IM	-	9 (2) ^†^	11 (3) ^†^	12 (2) ^†^
*HR*	OTM	108 ± 8	79 ± 18 ∗^†^	67 ± 18 ∗^†#^	59 ± 12 ∗^†#$^
	IM	111 ± 9	43 ± 6 ∗^†^	38 ± 3 ∗^†#^	38 ± 3 ∗^†#^
*SAP*	OTM	137 ± 15	136 ± 13 ^†^	142 ± 5 ^†^	144 ± 6 ∗^#^
	IM	136 ± 15	149 ± 7 ∗^†‡^	148 ± 6 ^∗†‡^	139 ± 7
*MAP*	OTM	110 ± 15	111 ± 16 ^†^	114 ± 5 ^†^	117 ± 6 ∗^#^
	IM	111 ± 16	122 ± 7 ∗^†‡^	120 ± 6 ∗^†‡^	112 ± 7
*DAP*	OTM	96 ± 15	97 ± 17 ^†^	100 ± 5 ^†^	104 ± 6 ∗
	IM	99 ± 16	108 ± 7 ^†‡^	107 ± 7 ^†‡^	99 ± 7
*f_R_*	OTM	63 ± 35	45 ± 30 ∗^†^	34 ± 16 ∗^†#^	30 ± 17 ∗^†#$^
	IM	70 ± 27	11 ± 3 ∗^†^	11 ± 3 ∗^†^	10 ± 3 ∗^†^
*BT*	OTM	38.8 ± 0.4	38.8 ± 0.5	38.8 ± 0.5	38.7 ± 0.4
	IM	39 ± 0.2	38.9 ± 0.2	38.8 ± 0.2	38.7 ± 0.3

∗ different from baseline; **^†^** difference between groups; ^#^ different from T10 within group; ^$^ different from T20 within group; ^‡^ different from T30 within group.
